# The *Cryptophlebia Leucotreta* Granulovirus—10 Years of Commercial Field Use

**DOI:** 10.3390/v7031284

**Published:** 2015-03-19

**Authors:** Sean D. Moore, Wayne Kirkman, Garth I. Richards, Peter R. Stephen

**Affiliations:** 1 Citrus Research International, PO Box 20285, Humewood, Port Elizabeth 6013, South Africa; E-Mails: wk@cri.co.za (W.K.); garth.richards@btinternet.com (G.I.R.); 2 Department of Zoology and Entomology, Rhodes University, PO Box 94, Grahamstown 6140, South Africa; 3 Citrus Research International, PO Box 28, Nelspruit 1200, South Africa; E-Mail: prs@cri.co.za

**Keywords:** *Cryptophlebia leucotreta* granulovirus, *Thaumatotibia leucotreta*, citrus, South Africa

## Abstract

In the last 15 years, extensive work on the *Cryptophlebia leucotreta* granulovirus (CrleGV) has been conducted in South Africa, initially in the laboratory, but subsequently also in the field. This culminated in the registration of the first CrleGV-based biopesticide in 2004 (hence, the 10 years of commercial use in the field) and the second one three years later. Since 2000, more than 50 field trials have been conducted with CrleGV against the false codling moth, *Thaumatotibia leucotreta*, on citrus in South Africa. In a representative sample of 13 field trials reported over this period, efficacy (measured by reduction in larval infestation of fruit) ranged between 30% and 92%. Efficacy was shown to persist at a level of 70% for up to 17 weeks after application of CrleGV. This only occurred where the virus was applied in blocks rather than to single trees. The addition of molasses substantially and sometimes significantly enhanced efficacy. It was also established that CrleGV should not be applied at less than ~2 × 10^13^ OBs per ha in order to avoid compromised efficacy. As CrleGV-based products were shown to be at least as effective as chemical alternatives, persistent and compatible with natural enemies, their use is recommended within an integrated program for control of *T. leucotreta* on citrus and other crops.

## 1. Introduction

The false codling moth, *Thaumatotibia leucotreta* (Meyrick) (Lepidoptera: Tortricidae), is one of the most important pests of citrus in Southern Africa [1,2]. A range of products have been tested for its control on citrus since 1926 [3]. These were reviewed by Moore [4]. However, since that time increased effort has been poured into the development of new technologies. The existing control measures for *T. leucotreta* are reviewed by Moore and Hattingh [5]. One of these is the *Cryptophlebia leucotreta* granulovirus (CrleGV) [4,6,7].

CrleGV was first described by Angelini *et al.* [8]. This isolate was obtained from infected *T. leucotreta* larvae from the Ivory Coast. *Thaumatotibia leucotreta* used to be known as *Cryptophlebia leucotreta*, hence, the name of the virus, but the host genus was changed in the late 1990s [9]. Angelini and Le Rumeur [10] stated that CrleGV contamination, if not curtailed, was capable of causing a laboratory-reared *T. leucotreta* culture to collapse. Incidentally, they also noted a cypovirus (CPV) infection in the laboratory culture. Another CrleGV isolate was obtained from diseased larvae, which were collected on the Cape Verde Islands [11]. Whitlock [12] was interested in the virus-like rods associated with CrleGV, which he isolated from a South African laboratory culture of the insect. A South African isolate was also obtained from larvae in a laboratory culture held by the Hoechst Corporation in Germany [13]. The South African isolate, the Ivory Coast isolate and the Cape Verde isolate can be clearly distinguished by restriction analysis [13]. Fritsch and Huber [14] made reference to biological and biochemical characterization of the three different isolates mentioned. Fragment patterns were determined by restriction enzyme analysis with *Eco*RI, *Bam*HI, and *Hind*III [15]. It was, thereby, demonstrated that all three isolates were distinct strains. Jehle *et al.* [13] constructed a restriction fragment map covering almost the entire genome of the Cape Verde isolate of CrleGV. The position of the *granulin* gene was identified by cross-hybridization with granulin coding fragments of *Cydia pomonella* GV (CpGV) [16]. The size of the viral genome was determined to be 112.4 kbp [13]. Its granulin amino acid sequence was compared to that of *Autographa californica* nucleopolyhedrovirus (AcNPV) polyhedrin, and other NPVs [17]. Jehle *et al.* [18] examined the genetic interaction between CrleGV and CpGV co-infecting larvae of *T. leucotreta*. In so doing, the genetic interaction of unmodified GVs was examined *in vivo* in order to assess possible risks of genetic exchange of modified baculoviruses. This work was based on the discovery that CpGV is cross-infectious for larvae of *T. leucotreta*, but is about 1000 times less virulent than the specific GV [19]. Subsequently, Lange and Jehle [20] sequenced and analyzed the entire CrleGV genome. The genome contained 110,907 bp and potentially encoded 129 predicted open reading frames (ORFs), 124 of which were similar to other baculovirus ORFs. A baculovirus chitinase gene was identified, but Lange and Jehle [20] concluded that it is most likely not functional, because its central coding region including the conserved chitinase active site signature was deleted. It was determined that CrleGV is indeed most closely related to CpGV, as revealed by genome order comparisons and phylogenetic analyses. However, the AT content of the CrleGV genome, which is 67.6% and the highest found so far in baculoviruses, differed by 12.8% from the AT content of CpGV. This resulted in a major difference in the codon usage of both viruses and may reflect adaptive selection constraints to their particular hosts.

Consequently, Reiser *et al.* [21] considered *T. leucotreta* as a suitable alternate host for mass production of CpGV for biological control purposes. This idea was apparently employed by Hoechst in Germany, but was unsuccessful, as CrleGV soon became the dominant virus in the culture [22]. This possibility is again being tested by Chambers [23], with renewed hope of success, due to improved techniques (based on qPCR) for rapid differentiation between the two viruses and, hence, establishment of virus purity [24].

Unlike the closely related CpGV, which has been widely tested since 1966 [25], culminating in the production of at least five commercial formulations [26], CrleGV was not exploited for the biological control of *T. leucotreta* on agricultural crops until 2004. Up to this time, only one record existed of a small-scale field trial with CrleGV, on citrus and Spanish pepper on the Cape Verde Islands [27].

In the last 15 years, extensive work on CrleGV has been conducted in South Africa, initially in the laboratory, but subsequently in the field too. Moore [4] described the discovery and development of a novel South African CrleGV isolate (CrleGV-SA) as a biological control agent for the management of *T. leucotreta* in South Africa. The granulovirus was identified from Goedehoop citrus insectary at Citrusdal, Western Cape, South Africa [4]. The CrleGV-SA isolate was subsequently characterized by Singh *et al.* [28]. Ludewig [29] attempted to induce a viral epizootic in larvae in a laboratory culture through stressing of the host, but concluded that this was not possible. He further concluded that this may be due to the *T. leucotreta* culture being virus-free, as PCR analysis of DNA extracted from asymptomatic larvae, sensitive down to 60 fg (480 genome copies of CrleGV), was unable to detect any CrleGV. However, Opoku-Debrah *et al.* [30] later succeeded in inducing outbreaks of CrleGV in five geographically distinct *T. leucotreta* laboratory cultures through overcrowding of larvae.

An artificial diet for the larval host, a rearing technique and a virus production system were developed [4,31]. Surface inoculation dose-response and time-response bioassays and detached fruit bioassays were conducted against *T. leucotreta* neonate larvae (the only instar that would be exposed to virus in the field) [7]. LC_50_ (the concentration required to kill 50% of the test insects) and LC_90_(the concentration required to kill 90% of the test insects) values were estimated to be 4.095 × 10^3^ occlusion bodies (OBs)/mL and 1.185 × 10^5^ OBs/mL, respectively. LT_50_ (time to kill 50% of the test insects) and LT_90_ (time to kill 90% of the test insects) values were estimated to be 4 days 22 h and 7 days 8 h, respectively, categorising the virus as a fast or type 2 granulovirus [32]. This was a clear indication that the virus was sufficiently virulent to warrant field trials. Consequently, extensive field trials were conducted [4,6,33], leading to registration of the biopesticide Cryptogran (River Bioscience, South Africa) [6]. Subsequently, a second CrleGV product, Cryptex (Andermatt Biocontrol, Switzerland) was registered for use against *T. leucotreta* in South Africa [34]. Registration of both products has been expanded to avocadoes and grapes [35]. Recently a third CrleGV product, Gratham (also a product of Andermatt Biocontrol, Switzerland), with specifications identical to Cryptex, has also been registered in South Africa. Consequently, CrleGV has been used commercially in the field for 10 years.

Goble [36] genetically and biologically characterized and compared the CrleGV isolates used in Cryptogran and Cryptex. Restriction analysis and partial amplifications of the *granulin* and *egt* genes, as amplicons of 690 bp and 1290 bp, revealed 99% and 98% nucleotide identities, respectively. The heterogeneity of the Cryptogran and Cryptex viral genotypes was further supported by significant differences in their biological activity determined by surface dose-response bioassays with neonate *T. leucotreta* larvae. Cryptogran was shown to be significantly more virulent (specifically the LC_90_) than Cryptex in dose-response bioassays. However, Opoku-Debrah *et al.* [37] subsequently showed that although this was significantly so in one case (comparing LD_50_ values of the isolates against neonate larvae from a regionally specific laboratory culture according to a protocol described by Pereira-da-Conceicoa *et al.* [38]), virulence is actually a very specific relationship between host and pathogen. Using seven CrleGV isolates and five *T. leucotreta* host populations, it was demonstrated that certain isolates were significantly more or less virulent against certain regionally distinct host populations [37].

The first reported case of insects developing resistance to a virus in the field was observed in *C. pomonella*, where field populations in Europe developed resistance to a Mexican isolate of CpGV (CpGV-M), after repeated field applications in organic orchards had failed [39,40,41,42]. In order to be prepared should a similar situation occur with *T. leucotreta* in South Africa, Opoku-Debrah *et al.* [30] bioprospected for new CrleGV isolates as possible alternatives to the existing ones used in the commercial formulations. This led to the isolation and genetic characterization of five novel CrleGV isolates. Single restriction endonuclease (REN) analysis of viral DNA and partial sequencing of *granulin* and *egt* genes and multiple alignments of nucleotide sequences were used to demonstrate these differences, leading to a proposal for two phylogenetic CrleGV-SA groups [30].

To date, 13 years of field trials with CrleGV have been conducted on citrus in South Africa. This amounts to well over 50 distinct field trials. This period includes 10 years of commercial field usage of CrleGV products (initially on citrus but also avocadoes and grapes), hence the title of this paper. Differentiation has been made between the early developmental work (with unformulated CrleGV) and trials with commercial products, due to the formulated preparations of the latter (which are proprietary). Apart from internal reports, theses [4,33] and one semi-popular paper [6], these trials have not previously been published.

Consequently, our primary objective in this paper is to report on a comprehensive, large and representative sample of these trials conducted on citrus in South Africa. This is therefore the first published account of the field use and efficacy of CrleGV and should be of great value to scientists and biocontrol practitioners throughout the region of distribution of *T. leucotreta*. Additionally, we have provided a mini-review in this introduction of all known studies to date conducted on CrleGV.

## 2. Results

### 2.1. Unformulated CrleGV

The first three trials reported were conducted with unformulated CrleGV. Results were surprisingly good, considering the lack of formulation, showing a reduction in *T. leucotreta* larval infestation of fruit of up to 60% in two of the trials and up to 82% in the other. However, these trials were conducted with extremely high rates of virus–up to more than 10^15^ OBs/ha. This is a lot higher than the rates used in all subsequent trials, which were conducted with formulated commercial CrleGV products and generally also in combination with adjuvants to enhance efficacy, thus permitting a reduction in concentration of OBs applied. The standard registered rates of existing commercial CrleGV products (in mL per L water) would facilitate application in the region of 6.6 × 10^12^ OBs/ha (Cryptex and Gratham) to 5 × 10^13^ OBs/ha (Cryptogran) [6].

**Figure 1:**
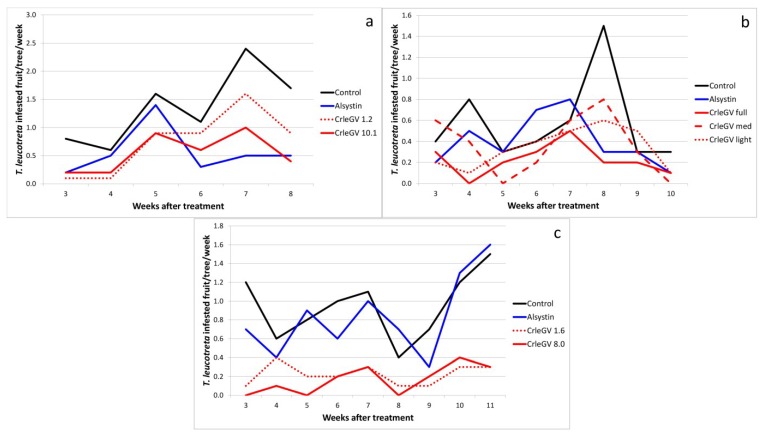
*Thaumatotibia leucotreta*-infested fruit per tree per week at (**a**) Sun Orange Farm. The trial was sprayed on 5 April 2001; (**b**) Vergenoeg Farm. The trial was sprayed on 14 March 2002; (**c**) Moosrivier Farm. The trial was sprayed on 16 January 2003. Concentrations given for CrleGV in (**a**) and (**c**) are × 10^14^ OBs per ha. (See Table 3 for full treatment application details; see Table 1 for means and standard errors for the full period of evaluation).

The first trial, conducted on Sun Orange Farm (Eastern Cape Province), used two rates, which differed almost 10-fold. Although there was no significant difference between the numbers of fruit infested with *T. leucotreta* larvae over the six week evaluation period, the higher rate resulted in significantly fewer fruit being infested than in the untreated control trees (reduced by 58.54%), whereas this was not the case for the lower rate (45.12% reduction in infestation; α = 0.05) (Figure 1a; Table 1).

In the second trial, conducted on Vergenoeg Farm (Eastern Cape Province), very similar rates of OBs per hectare were used for each of three CrleGV treatments (between 0.95 and 1.24 × 10^15^). The difference between the treatments was the volume of spray applied per hectare (or tree), varying between 15 and 27 L per tree (translating to between 8325 and 14,985 L per ha). We defined these as full, medium and light cover sprays. Although there was no significant difference between the numbers of fruit infested with *T. leucotreta* larvae over the eight week evaluation period, the full cover spray was the only treatment for which there were significantly fewer fruit infested than was the case in the untreated control trees (by 60.87%; α = 0.05) (Figure 1b; Table 1). This demonstrated the importance of not only applying an adequate concentration of virus particles, but also obtaining good coverage of the tree and its fruit.

**Table 1:** Treatment efficacy of CrleGV and CrleGV-based biopesticides and chemical standards against *T. leucotreta* in citrus field trials. Efficacy was measured by the reduction in numbers of fruit infested with *T. leucotreta* larvae relative to untreated control trees.

Site and year initiated	Treatment		Weeks evaluated	Fruit infested/tree/week		Reduction in infestation (%)
				Mean ^2^	SE	
	Product/s	Concentration/s ^1^	-	-	-	-
Sun Orange 2001	Control	-	6	1.37a	0.27	-
	CrleGV	1.2 × 10^14^	-	0.75ab	0.23	45.1
	CrleGV	1.0 × 10^15^	-	0.55b	0.14	58.5
	Alsystin	20 mL	-	0.57b	0.17	59.8
Vergenoeg 2002	Control	-	8	0.57a	0.15	-
	CrleGV full	9.5 × 10^14^	-	0.22b	0.05	60.9
	CrleGV med	1.2 × 10^15^	-	0.36ab	0.10	37.0
	CrleGV light	1.2 × 10^15^	-	0.34ab	0.07	41.3
	Alsystin	20 mL	-	0.40ab	0.09	30.4
Moosrivier 2003	Control	-	9	0.94a	0.12	-
	CrleGV	1.6 × 10^14^	-	0.22b	0.04	76.5
	CrleGV	8.0 × 10^14^	-	0.17b	0.05	82.3
	Alsystin	20 mL	-	0.83a	0.14	11.8
Carden 2003	Control	-	7	2.46a	0.28	-
	Cryptogran (blocks)	6.6 × 10^13^ (10 mL)	-	0.61b	0.10	75.0
	Cryptogran (single trees)	5.2 × 10^13^ (10 mL)	-	1.16c	0.05	52.9
	Control	-	17	1.84a	0.19	-
	Cryptogran (blocks)	6.6 × 10^13^ (10 mL)	-	0.56b	0.06	69.6
Bernol 2004	Control	-	5	2.56a	0.39	-
	Cryptogran	4.5 × 10^13^ (10 mL)	-	0.80bc	0.17	68.7
	Cryptogran	3.6 × 10^13^ (8 mL)	-	0.82bc	0.16	68.0
	Cryptogran	2.7 × 10^13^ (6 mL)	-	0.72bc	0.15	71.9
	Cryptogran	1.8 × 10^13^ (4 mL)	-	0.60b	0.13	76.6
	Cryptogran	9.0 × 10^12^ (2 mL)	-	1.12bc	0.21	56.2
	Cryptex	4.0 × 10^13^ (2.25 mL)	-	1.42c	0.46	44.5
Bernol 2005	Control	-	5	0.56a	0.16	-
	Cryptogran	5.4 × 10^13^ (10 mL)	-	0.34ab	0.12	39.3
	Cryptogran + molasses	5.4 × 10^13^ (10 mL) + 0.5	-	0.16b	0.06	71.4
	Cryptogran + molasses	5.4 × 10^13^ (10 mL) + 0.25	-	0.18b	0.09	67.9
Dunbrody 2006	Control	-	9	0.21a	0.05	-
	Cryptogran October, December, February	6.4 × 10^13^ (10 mL)	-	0.08b	0.04	63.2
	Cryptogran December	6.4 × 10^13^ (10 mL)	-	0.09b	0.03	57.9
	Cryptogran December, February	6.4 × 10^13^ (10 mL)	-	0.04b	0.02	78.9
Lone Tree 2007	Control	-	7	1.29a	0.22	-
	Cryptogran	6.1 × 10^13^ (10 mL)	-	0.90a	0.08	30.0
	Cryptogran + molasses	6.1 × 10^13^ (10 mL)	-	0.17b	0.02	86.7
Lone Tree 2008	Control	-	6	0.62a	0.05	-
	Cryptogran	4.2 × 10^13^ (10 mL)	-	0.37b	0.10	40.5
	Cryptogran + molasses	4.2 × 10^13^ (10 mL)	-	0.18c	0.05	70.3
	Cryptex + molasses	8.0 × 10^12^ (3.3 mL)	-	0.30bc	0.04	51.3
Welegelegen 2009	Control		9	0.12a	0.03	-
	Cryptogran December, March	2.8 × 10^13^ (10 mL)	-	0.03b	0.01	76.9
	Cryptogran March	2.8 × 10^13^ (10 mL)	-	0.01b	0.01	92.3
Far Away 2010	Control	-	5	1.78a	0.23	-
	Cryptogran	3.0 × 10^13^ (10 mL)	-	1.16a	0.14	34.8
	Cryptogran + molasses	3.0 × 10^13^ (10 mL)	-	0.82b	0.09	53.9
	Cryptex	3.9 × 10^12^ (3.3 mL)	-	1.24a	0.14	30.3
	Cryptex + molasses	3.9 × 10^12^ (3.3 mL)	-	1.04a	0.14	41.6
	Delegate	20 g	-	0.80b	0.08	55.0
	Alsystin	20 mL	-	0.68b	0.09	61.8
Bernol 2011	Control	-	7	0.16a	0.05	-
	Cryptogran	6.7 × 10^13^ (10 mL)	-	0.10a	0.04	36.4
	Cryptogran + molasses	6.7 × 10^13^ (10 mL) + 0.25	-	0.04b	0.02	72.7
	Cryptex	8.8 × 10^12^ (3.3 mL)	-	0.11a	0.04	27.3
	Cryptex + molasses	8.8 × 10^12^ (3.3 mL) + 0.5	-	0.10a	0.04	36.4
Far Away 2013	Control	-	6	0.23a	0.05	-
	Cryptogran + molasses	3.2 × 10^13^ (10 mL)	-	0.10b	0.04	57.1
	Cryptex	4.3 × 10^12^ (3.3 mL)	-	0.15a	0.04	35.7
	Runner	60 mL	-	0.07b	0.02	71.4
	Delegate	20 g	-	0.07b	0.03	71.4

^1^ CrleGV given in approximate OBs/ha, followed in brackets in concentration per 100 L water for formulated products; molasses given in percentage concentration (molasses is only listed if CrleGV treatments were applied both with and without molasses in the same trial; concentration is only given if more than one concentration was used in a trial); chemical insecticides given per 100 L water; ^2^ Values per trial that are followed by the same letter are not significantly different (Fisher LSD multiple range test; α = 0.05).

In the third trial, conducted on Moosrivier Farm (Mpumalanga Province), two rates of CrleGV, with a five-fold difference in concentration, were applied. Both treatments were extremely effective—producing a 76% and an 82% reduction in infestation, respectively, over the nine-week evaluation period (Figure 1c; Table 1).

### 2.2. CrleGV Efficacy in Block vs. Single Tree Treatments

**Figure 2:**
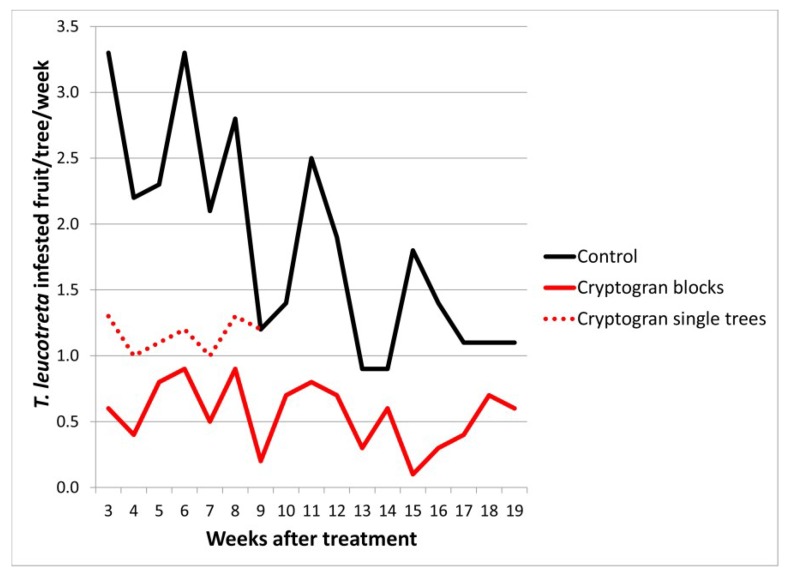
*Thaumatotibia leucotreta*-infested fruit per tree per week at Carden Farm. The trial was sprayed on 3 December 2003. (See Table 3 for full treatment application details; see Table 1 for means and standard errors for the full period of evaluation).

In only one trial was the efficacy of CrleGV applied in blocks compared to that applied to single trees (in a randomized format). This was conducted with Cryptogran on Carden Farm (Eastern Cape Province) in 2003. The blocks used in the trial consisted of 82 trees each and measured 0.15 ha in size. The single-tree treatments were applied with handguns at an average of more than 20 L of spray mix per tree and the blocks were treated with three quarters of this volume using tractor-drawn oscillating tower mistblowers. Not only was the volume applied to the single trees higher, but the handgun application would have facilitated more targeted and hence better coverage than the automatic spray machinery. Despite this, treatment efficacy in the blocks was dramatically superior to that on the single trees. This pertained to both level of efficacy (there was a significant difference in numbers of fruit infested between the two treatments (α = 0.05), translating into a 75% compared to a 53% reduction in *T. leucotreta* infestation over seven weeks) and duration of efficacy (mean of 70% efficacy recorded at 17 weeks after treatment in blocks, whereas efficacy on single trees disappeared after seven weeks) (Figure 2; Table 1). The results of this trial are also reported in Moore *et al.* [6].

### 2.3. Effect of Molasses as an Adjuvant

A total of five of the trials conducted investigated the value of adding molasses to either Cryptogran or Cryptex.

**Figure 3:**
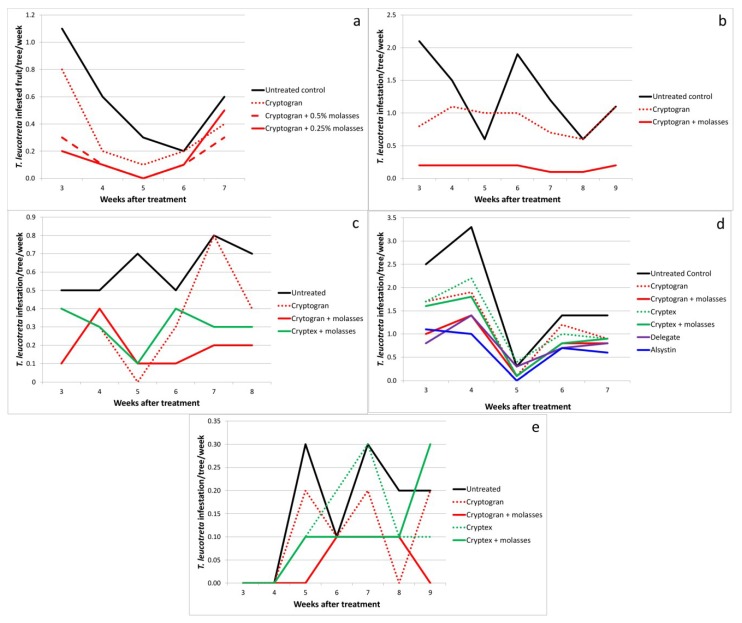
*Thaumatotibia leucotreta*-infested fruit per tree per week at (**a**) Bernol Farm. The trial was sprayed on 22 March 2005; (**b**) Lone Tree Farm. The trial was sprayed on 10 December 2007; (**c**) Lone Tree Farm. The trial was sprayed on 10 December 2008; (**d**) Far Away Farm. The trial was sprayed on 7 December 2010; (**e**) Bernol Farm. The trial was sprayed on 1 December 2004. (See Table 3 for full treatment application details; see Table 1 for means and standard errors for the full period of evaluation).

In the second trial conducted on Bernol Farm (Eastern Cape Province) (in 2005), Cryptogran was applied with 0.5% molasses (500 mL per 100 L water), as it was registered at the time [6]. Two other treatments were included: Cryptogran without molasses (in order to determine whether molasses did indeed improve efficacy) and Cryptogran with a reduced concentration of molasses (0.25%) and a surfactant. *Thaumatotibia leucotreta* larval infestation of fruit was not significantly different between the Cryptogran (alone) treatment and the untreated control, whereas significantly fewer fruit were infested in both Cryptogran and molasses treatments than in the untreated control (α = 0.05) (infestation reduced by 71% and 68%) (Figure 3a; Table 1).

Two trials with molasses and CrleGV were conducted on Lone Tree Farm (Eastern Cape Province) in subsequent years, 2007 and 2008. By 2007 the registered rate of molasses with Cryptogran had been reduced from 0.5% to 0.25% and included the addition of a surfactant. This new registration was compared with Cryptogran without any additives. Cryptogran on its own reduced *T. leucotreta* infestation by only 30% (number of fruit infested not being significantly different from the untreated control), whereas Cryptogran with the additives reduced infestation by almost 87% (Figure 3b; Table 1). In the 2008 trial, although both Cryptogran without molasses and Cryptogran with molasses resulted in significantly fewer fruit being infested with larvae than in the untreated control, Cryptogran with molasses added resulted in significantly fewer fruit being infested than did Cryptogran alone (α = 0.05) (translating into a 70% and a 40% reduction in infestation, respectively, relative to the control) (Figure 3c; Table 1).

This trend was confirmed in the trials conducted on Far Away Farm (Eastern Cape Province) in 2010 and Bernol Farm in 2011. In both trials, both Cryptogran and Cryptex were applied with and without molasses. Cryptogran was applied with 0.25% molasses and a surfactant, as registered. Cryptex was applied with 0.5% molasses as this was its original registration. However, this was subsequently changed in 2012 when Cryptex was registered without the need for the addition of molasses. At Far Away Farm, the only CrleGV treatment for which infestation of fruit was significantly lower than the untreated control was Cryptogran with molasses (α = 0.05) (Figure 3d; Table 1). This treatment was also significantly more effective, as measured by comparing fruit infestation, than Cryptogran alone. Although Cryptex with molasses was not significantly more effective than Cryptex alone, its superior efficacy was notable. The same trend was noted with both products at Bernol Farm (Figure 3e; Table 1).

### 2.4. Dose Rate

**Figure 4:**
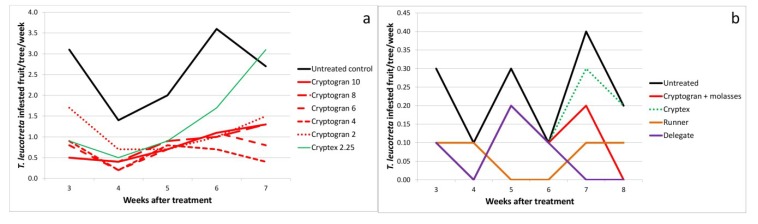
*Thaumatotibia leucotreta*-infested fruit per tree per week at (**a**) Bernol Farm. The trial was sprayed on 1 December 2004. (Concentrations given in mL/100 L water); (**b**) Far Away Farm. The trial was sprayed on 24 April 2013. (See Table 3 for full treatment application details; see Table 1 for means and standard errors for the full period of evaluation).

Despite Cryptogran being registered in 2004 at a concentration of 10 mL per 100 L water, a trial was conducted on Bernol Farm in 2004 to determine whether any dose response could be detected at a range of concentrations from 10 mL down to 2 mL per 100 L water. Additionally, Cryptex, which was not yet registered when the trial was conducted, was included at the rate proposed for registration by the suppliers at that time, *i.e.*, 200 mL of product per ha, which was equivalent to 2.25 mL per 100 L water. As Cryptex was a more dilute preparation of virus than Cryptogran, this rate was approximately equivalent to 1 mL of Cryptogran per 100 L water. All treatments resulted in a significantly fewer fruit being infested than in the untreated control (Figure 4a; Table 1). As would be expected, the lowest concentration treatment (Cryptex) was the least effective, but surprisingly, the most effective treatment was 4 mL Cryptogran per 100 L water (the only treatment which was significantly more effective than Cryptex), indicating that the registered Cryptogran rate (10 mL per 100 L water) could be substantially reduced without an immediate loss of efficacy.

A dose-response was not observed in this dose trial on Bernol Farm. However, the large difference in concentration between the four highest rates and the lowest rate was large enough for there to be a discernible difference in efficacy. This is confirmed by the results from the first trial conducted at Sun Orange Farm, where the 10-fold difference in CrleGV concentrations was large enough to lead to a marked difference in efficacy (only the higher dose with significantly fewer infested fruit than the untreated control) (Figure 1a; Table 1).

A further four trials compared the efficacy of Cryptogran and Cryptex: Lone Tree Farm, 2008; Far Away Farm, 2010 and 2012; and Bernol Farm, 2011. In all four trials Cryptogran was more effective than Cryptex (Table 1). This was particularly a reliable assessment where the two products were compared either both with molasses or both without molasses. The difference in numbers of fruit infested was significant at Bernol Farm in 2011 (α = 0.05), where Cryptogran plus molasses reduced *T. leucotreta* infestation by 73%, whereas Cryptex plus molasses only reduced infestation by 36% (Figure 3e; Table 1).

Only in the last trial–Far Away Farm in 2012–was Cryptogran with molasses compared to Cryptex without molasses as by this time Cryptex was no longer registered to be used with molasses. Here Cryptogran was significantly more efficacious than Cryptex, measured by numbers of fruit infested (α = 0.05) and translating into a 57% and a 36% reduction in infestation, respectively (Figure 4b; Table 1). However, this difference in efficacy may not only have been a result of the difference in OB concentration, but also the non-inclusion of molasses with Cryptex.

### 2.5. CrleGV Spray Programs

**Figure 5:**
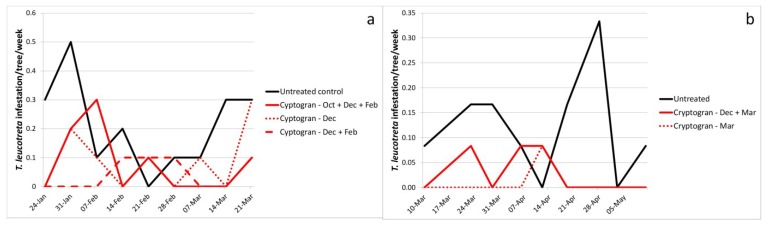
*Thaumatotibia leucotreta*-infested fruit per tree per week at (**a**) Dunbrody Farm. The trial was sprayed on 26 October and 5 December 2006 and 5 February 2007; (**b**) Welgelegen Farm. The trial was sprayed on 8 December 2009 and 15 March 2010. (See Table 3 for full treatment application details; see Table 1 for means and standard errors for the full period of evaluation).

In 2006 a trial was conducted on Dunbrody Farm (Eastern Cape Province) to compare the efficacy of three different Cryptogran programs. All programs (one, two and three applications) resulted in significantly fewer *T. leucotreta* infested fruit than in the untreated control (α = 0.05), translating into a reduction in fruit infestation of 58%, 79% and 63%, respectively (Figure 5a; Table 1). Although there was no significant difference in numbers of fruit infested between the one (December) and two (December and February) spray programs, the recorded improvement in efficacy with the two-spray program indicated that this may be the superior option. The three-spray program included an early spray in October, which did not appear to improve overall efficacy. *Thaumatotibia leucotreta* activity is usually very low at this time (spring), particularly in Navel oranges [1,4,43] and, therefore, a treatment this early may often be superfluous.

The only other trial in which more than one CrleGV treatment was applied on the same treatment block was at Welgelegen Farm (Mpumalanga Province). Here the efficacy of a single spray of Cryptogran in March on Marsh grapefruit was compared with that of a double spray (December and March). Surprisingly, number of fruit infested for the single spray was slightly, but not significantly, lower than for the double spray (92% reduction in infestation relative to the untreated control, compared to 77% reduction) (Figure 5b; Table 1). This may either be an indication that *T. leucotreta* only begins attacking Marsh grapefruit in the latter half of the season as the fruit begins to ripen (which conforms to anecdotal reports) or that *T. leucotreta* pressure was so low (only a mean of 0.12 infested fruit per tree per week in the untreated control, therefore, lower than at any of the other trial sites) that a single treatment was sufficient. Both of these theories are well supported [2]. However, this would certainly not be the case for more susceptible citrus types, such as Navel oranges, which are known to be subject to heavier attack from earlier in the growing season [44].

### 2.6. Comparison with Chemical Insecticides

In five out of the 13 trials a chemical standard was included. In two out of the four trials in which triflumuron (Alsystin) was used, CrleGV performed similarly to triflumuron, whereas in the other two trials, CrleGV outperformed triflumuron. This poor efficacy may have been a case of the well documented triflumuron resistance development by *T. leucotreta* [45], something which appears to occur after six to seven years of regular use.

Spinetoram (Delegate) was compared to CrleGV in two trials, with similar or slightly better efficacy to the best CrleGV treatment. Methoxyfenozide (Runner) was used in one trial, with slightly better efficacy to the best CrleGV treatment. None of these differences were statistically significant (α = 0.05).

## 3. Discussion

*Thaumatotibia leucotreta* is an important pest in the Southern African citrus industry [1,2]. It is extremely important to control it effectively, particularly due to its endemism to Africa [4] and the exporting of around 70% of South Africa’s fresh citrus to foreign markets [46]. CrleGV-based biopesticides, such as Cryptogran and Cryptex, have proven to be effective tools for aiding in suppressing and controlling this cryptic pest.

Although a single-tree randomized block trial layout lends itself to more accurate and reliable comparison of treatment efficacy, as both the randomization and the use of a relatively small homogenous area manage for any possible variation very well (which is why the design was so often used in trials), it was shown in the trial conducted at Carden Farm in 2003, that CrleGV treatments applied to blocks of trees will provide far more effective control of *T. leucotreta*. This is because in a single tree layout, there is very little or no buffer against *T. leucotreta* pressure from outside of the trial area. Additionally, recolonization of *T. leucotreta* from adjacent or nearby untreated trees (or trees treated with less effective or ineffective treatments) will occur immediately on breakdown of a treatment. The semi-commercial block format used for testing CrleGV therefore provided a more accurate measurement of the true potential of CrleGV to control *T. leucotreta* in citrus under commercial conditions.

In four out of the 13 trials presented in this study, such a block format was used. Cryptogran succeeded in reducing *T. leucotreta* infestation by between 58% and 92% (average of 72%) in these treatments (considering only those concentrations high enough to give optimal efficacy). One might argue that any level of control less than a percentage which is in the high 90s against a potentially phytosanitary pest is inadequate. However, Moore and Hattingh [5] point out that it is essential that *T. leucotreta* be controlled using an integrated suite of control options. Therefore, the efficacy of a single product application should not be judged in isolation but as part of a whole. For example, if a farmer uses five different control practices against *T. leucotreta* (these could for argument’s sake be any of orchard sanitation, parasitoid conservation (or augmentation), CrleGV sprays, mating disruption and a chemical spray) and these very conservatively each provide around 50% control, the combined efficacy would be in the region of 97%. In reality, treatment efficacy would generally be expected to be well above this level for most products and technologies [5].

Furthermore, CrleGV is completely harmless to beneficial insects. Grout *et al.* [47] conducted a series of bioassays with Cryptogran field-weathered residues on citrus leaves against four key natural enemies of citrus pests: *Chilocorus nigritus* (Fabricius) (Coleoptera: Coccinellidae), *Aphytis lingnanensis* Compere (Hymenoptera: Chalcididae), *Coccidoxenoides perminutus* (Timberlake) (Hymenoptera: Encyrtidae) and *Trichogrammatoidea cryptophlebiae* (Nagaraja) (Hymenoptera: Trichogrammatoidea). These were conducted according to the protocol established and described by Hattingh *et al.* [48]. They concluded that Cryptogran is probably the softest pesticide that they had tested with regard to its toxicity to natural enemies, as its overall impact ratings were below 10% for the natural enemies tested. Consequently, Cryptogran was categorised as “Harmless” against natural enemies considered important in the citrus ecosystem.

This would obviously be in contrast to the chemical alternatives for *T. leucotreta*, which would certainly have a far more adverse effect against natural enemies, including those which attack *T. leucotreta*, than would CrleGV. In total at least 17 parasitoids of *T. leucotreta* have been recorded [1,2,4,49]. The most important of these is the egg parasitoid, *T. cryptophlebiae* [2]. It can dramatically reduce *T. leucotreta* levels in citrus orchards, either by inundative augmentation [50] or conservation [51]. If one couples this with the fact that the chemical alternatives tested did not perform better than did CrleGV, one can only conclude that CrleGV is a very attractive option for *T. leucotreta* control.

Only one previous record of a field trial with CrleGV against *T. leucotreta* exists. This was a small-scale field trial on citrus and Spanish pepper on the Cape Verde Islands [27]. Concentrations of 10^8^ and 10^9^ OBs/mL were used, and only skimmed milk powder and a wetting agent were added to the virus suspensions. *Thaumatotibia leucotreta* damage was reduced by 77% in citrus and 65% in Spanish pepper [27]. Although these concentrations used were extremely high compared to the registered concentrations with Cryptogran and Cryptex (5 × 10^6^ and 6.6 × 10^5^ OBs/mL, respectively), efficacy was not dissimilar to that reported in our studies. This may be because milk powder is not as effective as molasses at enhancing the efficacy of CrleGV in the field [33].

In truth, it appears that from trials conducted in this study that compared different dose rates of CrleGV, it may be possible to further reduce the amount of virus applied, without loss of efficacy. For example, in the trial conducted on Bernol Farm in 2004, although the lowest Cryptogran rate (2 mL per 100 L water) was the least effective rate used, the difference in efficacy was not statistically significant. Nevertheless, this may be an indication that the application rate of CrleGV can be dropped to around 2 × 10^13^ OBs per ha without any immediate loss of efficacy. However, although this reduction in application rate from the registered Cryptogran rate may not reduce immediate efficacy, it may reduce residual efficacy, as breakdown (mainly due to ultraviolet (UV) irradiation) to below the critical minimum level of viable OB density on the tree for optimal efficacy, would then be reached sooner. It is not surprising that a dose-response was not observed in the trial on Bernol Farm, as dose-responses to baculoviruses are not easily observed in the field [52]. Nevertheless, the large difference in concentration between the four highest rates and the lowest rate was large enough for there to be a discernible difference in efficacy.

It was noted that Cryptogran was consistently more effective than Cryptex. The reason for this consistent difference in efficacy must almost certainly be a result of the difference in concentration of OBs applied, being 7.6 times higher with Cryptogran (based on the registered concentrations of the two products). However, an additional explanation is a possible differential susceptibility of the local population of *T. leucotreta* to the isolates of virus present in both commercial products. Most of the field trials were conducted in the Addo region of Sundays River Valley in the Eastern Cape Province. Opoku-Debrah *et al.* [37] demonstrated in laboratory bioassays that the CrleGV isolate in Cryptogran was significantly more virulent to neonate *T. leucotreta* larvae from this region than was the CrleGV isolate in Cryptex. Cryptex required an estimated mean of 2.58 OBs per larva to elicit 50% mortality (LD_50_) in a given population as opposed to 1.02 OBs required for Cryptogran. LD_90_ for Cryptex and Cryptogran were 669 and 273 OBs per larva, respectively [37].

Despite a number of general trends being observed in these field trials, it would be prudent to conduct a meta-analysis in order to confirm patterns [53]. However, this would be superfluous with the relatively small number field trials reported here. A meta-analysis should be conducted on the full complement of more than 50 field trials and therefore warrants a separate study.

Considering the dearth of other CrleGV field trials, it is interesting to compare our results and experiences with those of the closely related system of the *Cydia pomonella* granulovirus (CpGV) against the codling moth, *Cydia pomonella* (Lepidoptera: Tortricidae), in apples (*i.e.*, both cryptic tree fruit pests from the moth family, Tortricidae). Extensive field studies have been conducted with this system since 1966 [25]. Lacey *et al.* [26] listed numerous different field studies with CpGV, conducted on all continents of the world. There are therefore many examples that can be quoted.

Huber and Dickler [54] tested CpGV in a commercial apple orchard for two years and compared it to organophosphate insecticides. They were able to achieve a 44%–85% reduction in injury to apples as opposed to a 72%–98% reduction with the use of chemical applications. Later studies by Jaques *et al.* [55] showed that the use of CpGV could reduce *C. pomonella* deep-entry damage to apples by 40%–83% compared to the respective control plots. In some of their trial data the protection of fruit by CpGV unexpectedly exceeded that of an organophosphate insecticide. Sheppard and Stairs [56] tested a range of doses from 10^7^ to 10^9^ OBs/tree. All the doses tested had a similar effect on the reduction of infestation but it was found that with the higher dosages there was a larger reduction in larval population as they entered the fruit. Falcon *et al.* [25] reported a 90% reduction in shallow entries. Stará and Kocourek [57] tested various concentrations of CpGV ranging from 0.5 to 1 × 10^13^ OBs/ha, as well as varying numbers of applications per season. They succeeded in reducing the *C. pomonella* population by 75%–96% compared to 91%–97% achieved with teflubenzuron. Arthurs *et al.* [58] tested three concentrations of CpGV against high *C. pomonella* populations, resulting in 81%–99% larval mortality in fruit and a reduced number of mature larvae collected in tree bands by 54%–98%. However, these studies showed that CpGV was more effective at reducing the *C. pomonella* population density than reducing fruit injury. Glen and Clark [59] found that different treatments of CpGV did not significantly affect the survival of the neonate larvae before they entered the fruit. In their first trials, 49% of larvae survived long enough to cause recognizable damage to the fruit. In a subsequent experiment 69% of larvae produced damage to the fruit irrespective of the treatment applied. However, it was noted that the neonate larvae usually died shortly after entering treated fruit. This highlighted a potential shortcoming of CpGV, namely its speed of kill.

Efficacy recorded in our trials with CrleGV against *T. leucotreta*, fell within the range reported for trials with CpGV against *C. pomonella*. However, unlike CpGV (against *C. pomonella* on apples), speed of kill does not appear to be a shortcoming with CrleGV (against *T. leucotreta* on citrus). Negligibly few dead (virus infected) larvae were found in fruit that had been treated with CrleGV. A first instar larva takes approximately four days to penetrate through the rind and albedo of a citrus fruit [7]. If the larva dies or if its behaviour changes (and it reverses out of the fruit) before it manages to penetrate through the albedo into the flesh of the fruit, the damage to the fruit may be insignificant, meaning that the fruit will not decay and the minute blemish on the rind will not downgrade the fruit for export [7], unlike an apple. This behaviour is typical of symptomatically baculovirus-infected lepidopteran larvae [32].

Another drawback with CpGV in the field appears to be its rapid breakdown due to UV degradation. Half-life of CpGV in the field is generally estimated to be between two and three days [60,61,62,63,64,65]. Glen and Payne [66] showed that although CpGV infectivity was reduced by half in three days, some activity persisted for as long as four to eight weeks after spraying. Arthurs and Lacey [52] reported that early season applications of label rates of three CpGV products remained highly effective for the first 24 h (producing 94% larval mortality) and moderately effective after 72 h (71% mortality), declining to 50% of its original value after eight days (early summer) during dry sunny conditions. However, some activity remained for up to 14 days, suggesting prolonged survival of the virus in UV-protected locations, such as the calyx of fruit. The decline to 50% activity was more rapid (four days) in mid-summer. Consequently, the recommended application intervals for CpGV against *C. pomonella* range from 7 to 14 days [52,54,57,58,62,67,68].

As with the slow speed of kill, so too does it appear that rapid breakdown of virus is not a problem with CrleGV on citrus, as it is with CpGV on apples. Particularly the trial conducted at Carden Farm in 2003 demonstrated efficacy of almost 70% recorded at 17 weeks after application. This was a minor decline in efficacy from the 81% recorded at three weeks after application. Fritsch and Huber [69] estimated the half-life of CrleGV in the field to be two to three days, therefore similar to CpGV. However, Moore [4] demonstrated that although CrleGV appeared to break down to less than 50% of its original activity within 3–6 days on the northern (sunny) sides of citrus trees, at 21 days after application, efficacy had not yet dropped to this level on the southern (shady) sides of trees. More recently, Mwanza [70] confirmed this phenomenon, in an attempt to determine CrleGV reapplication frequency required in the field. He established that at 21 days after application to citrus trees in the field, LD_50_ of CrleGV (against neonate *T. leucotreata* larvae) recovered from the northern sides of trees was 15 times higher than from the southern sides of trees. By 28 days after application, virulence of CrleGV on the northern sides of trees was indeterminable, whereas on the southern sides of trees, there was still a clear dose response.

Moore *et al.* [6] surmised that there are four reasons for the protracted CrleGV persistence recorded on citrus. Firstly, a citrus tree provides substantial shading and therefore protection of virus against UV inactivation–more so than probably any other crops on which viruses have been tested for pest control. Secondly, it has been observed that during most of the growing season, the vast majority of *T. leucotreta* larvae penetrate a Navel orange through its navel end. It is precisely here that CrleGV could be well protected against sunlight and possibly even rainfall. Thirdly, *T. leucotreta* takes a long time to recolonise an area, even once the efficacy of a spray might have expired. This slow migration is confirmed by Timm *et al.* [71] and Stotter *et al.* [43]. Lastly, as CrleGV would have little, if any, detrimental impact on the highly effective and naturally occurring egg parasitoid, *T. cryptophlebiae*, this biocontrol agent could aid in maintaining control of *T. leucotreta* once virus was no longer effective.

Despite all of these positives, there are a number of challenges that may occur and should be addressed in future research. The risk of development of resistance by the target pest to CrleGV has been mentioned. This concern is based on the experiences with CpGV and *C. pomonella* in Europe [39,40,41,42]. However, as CpGV is recommended to be applied every 7 to 14 days [45,54,57,58,62,67,68] and CrleGV is applied far less frequently, the risk of resistance development must surely be less. Nevertheless, the study initiated on identification of novel isolates [30] should be continued and expanded. The potential for resistance can be tested in the laboratory by inducing resistance under selection pressure in subsequent generations, such as was achieved with *Phthorimaea operculella* (Zeller) to PhopGV [72,73,74] and *Anticarsia gemmatalis* (Hubner) to AgMNPV [75]. The ability of novel CrleGV isolates to overcome resistance can then be tested against these resistant individuals in laboratory assays. The genetic basis for this ability to overcome resistance should then be determined. For example, it has been ascertained that the viral gene pe38 is not only essential for the infectivity of CpGV but it is also the key factor in overcoming CpGV resistance in codling moth [76,77].

As Opoku-Debrah *et al.* [37] has already determined that certain CrleGV isolates are significantly more virulent than others against laboratory cultures of certain regionally distinct *T. leucotreta* populations, this study should be extended to the field (using isolates at equivalent dose rates) to determine if these differences do indeed translate into practice–something which we may already have observed with the differences in efficacy between the two main commercial preparations in the Eastern Cape Province. This may lead to the development of regionally appropriate commercial preparations of CrleGV. This is a possibility that should also be investigated for other baculovirus-host systems. For example, similar differences have been recorded in the laboratory for both virulence of different CpGV genomes [78] and susceptibility of different *C. pomonella* populations [79].

Another challenge that warrants attention is that of UV protection. Although it has been stated that protection from UV by the architecture of a citrus tree is superior to that of an apple tree, there must be exceptions. For example, a young small tree will be far sparser than a mature tree and will thus provide less shading. Additionally, cultivars other than Navel oranges do not possess a navel end in which OBs can be protected against direct sunlight and where *T. leucotreta* larvae will preferentially penetrate. Although numerous studies have demonstrated significant protection of baculoviruses under laboratory conditions (e.g., [80,81,82,83,84]), there is as yet insufficient evidence that this makes a substantial difference in the field under commercial practices (e.g., [85]). Consequently, examination of these published formulations with CrleGV, all the way up to full field trials is justified. Additionally, it can be assumed that effective commercial formulations will be kept proprietary. Consequently, sophisticated research on novel and effective formulations should be conducted outside of the commercial sector in order that this information can be made available to scientists and practitioners in the field.

## 4. Material and Methods

Of the more than 50 field trials that were executed over a 15 year period, only a sample has been presented here. Trials where pest levels were too low to obtain a reliable result and trials which were designed to investigate factors other than efficacy have not been included in this paper. Highly experimental treatments that were used in trials or treatments that are superfluous to this paper have also been omitted.

### 4.1. Source of CrleGV

For trials conducted from 2001 to 2003, CrleGV was produced *in vivo* in the laboratories of Citrus Research International in Port Elizabeth. This production process is described by Moore [4]. For trials conducted from 2004 onwards, commercially available products were used in trials. These were Cryptogran and Cryptex. Cryptogran is bottled at a nominal concentration of 5 × 10^10^ OBs/mL and registered to be applied at a concentration of 10 mL per 100 L water, whereas Cryptex is bottled at a nominal concentration of 2 × 10^10^ OBs/mL and registered to be applied at a concentration of 3.3 mL per 100 L water.

### 4.2. Trial Sites

All trials were conducted in established citrus orchards on commercial farms in South Africa (Table 2). Nine of the 13 trials were conducted in the Sundays River Valley of the Eastern Cape Province. One of the trials was conducted in the Gamtoos River Valley in the same province. The other two trials were conducted in the Mpumalanga Province.

**Table 2:** Details of citrus trial sites where CrleGV was field tested against *T. leucotreta*.

Years (Citrus season) in which trial was conducted	Farm name	Coordinates	Cultivar	Tree age (years)	Trees/ha	Trial layout
2000/01	Sun Orange	33°28'06"S 25°39'00"E	Palmer Navel	21	383	STRB
2001/02	Vergenoeg	33°45'45"S 24°48'59"E	Robyn Navel	17	555	STRB
2002/03	Moosrivier	25°01'24"S 29°22'22"E	Robyn Navel	15	340	STRB
2003/04	Carden	33°28'13"S 25°41'23"E	Palmer Navel	11	555	STRB SCB
2004/05	Bernol	33°28'26"S 25°36'43"E	Washington Navel	6	595	SCB
2004/05	Bernol	33°28'26"S 25°36'43"E	Palmer Navel	7	555	STRB
2006/07	Dunrody	33°27'59"S 25°31'30"E	Lane Late Navel	10	833	SCB
2007/08	Lone Tree	33°51'56"S 25°41'31"E	Palmer	8	555	STRB
2008/09	Lone Tree	33°51'56"S 25°41'31"E	Palmer Navel	9	555	STRB
2009/10	Welgelegen	25°27'51"S 31°53'02"E	Turkey Valencia	8	555	SCB
2010/11	Far Away	33°29'07"S 25°40'34"E	Newhall	3	555	STRB
2011/12	Bernol	33°28'26"S 25°36'43"E	Palmer Navel	6	555	STRB
2012/13	Far Away	33°29'07"S 25°40'34"E	Witkrans Navel	5	555	STRB

STRB = single-tree randomised block; SCB = semi-commercial block.

### 4.3. Trial Layout

Trials were either laid out in single-tree randomised block format, replicated 10 or 12 times or in semi-commercial block format with two replicates per site (Table 2). If trees were considered too close to one another that spray drift from spraying an adjacent tree was a risk, then every second tree was used as an unsprayed buffer tree.

In semi-commercial trials, block (replicate) size ranged between 0.15 ha (82 trees) and 0.18 ha (150 trees).

### 4.4. Treatment Application

All CrleGV treatments were applied at or after sunset in order to avoid any immediate breakdown of virus from UV radiation. All sprays were applied as high volume full cover film sprays as defined by Grout [86], unless stated otherwise, as in the case of only one of the trials. All single-tree randomized block trials were applied using a high-pressure hand-gun spray applicator powered by a Honda 250 cc engine and towed through orchards using a four wheel drive utility vehicle. In all cases, pump pressure was set at 20 bar and 2 mm orifice-diameter spray nozzles were used on spray guns. All semi-commercial block trials were applied using a tractor-drawn, power take-off (PTO)-driven high profile oscillating tower mistblower, using 2 mm nozzles with TeeJet “56” cores and set at 20 bar pressure. As the mistblower used belonged to the farm on which each trial was conducted, the make of mistblower differed from trial to trial.

### 4.5. Trial Details

All relevant details of trial application are provided (Table 3). Where CrleGV was applied with a surfactant, this was either Agral 90 (alkylated phenol-ethylene oxide; Plaaskem, Boksburg, GP, South Africa) at 18 mL/100 L water or Break-Thru S240 (polyether trisiloxane; Evonik Africa, Midrand, GP, South Africa) at 5 mL/100 L water. Chemical standards were included in some of the trials for comparison. These were: Alsystin 480 SC (triflumuron; Bayer CropScience, Monheim am Rhein, NRW, Germany), which was sprayed at 20 mL/100 L water; Delegate 250 WG (spinetoram; Dow AgroSciences, Indianapolis, IN, USA), which was sprayed at 20 g/100 L water; and Runner 240 SC (methoxyfenozide; Dow AgroSciences, Indianapolis, IN, USA), which was sprayed at 60 mL/100 L water. All of these are the registered concentrations. Trials which included these chemical standards are not indicated in Table 3. However, their inclusion and results are given in the figures and table for Results.

**Table 3:** CrleGV application details in field trials conducted against *T. leucotreta* in citrus orchards.

Farm	Treatment application date	Type of CrleGV	Concentration for commercial products (mL/100 L Water)	OBs/ha	Concentration of molasses (%) ^1^	Volume applied (mean L/tree)	Spray method
Sun Orange	5 April 2001	Unformulated	-	1.22 × 10^14^	-	38.3	Handguns
-	-	-	-	1.01 × 10^15^	-	-	-
Vergenoeg	14 March 2002	Unformulated	-	9.52 × 10^14^	-	27.0	Handguns
-	-	-	-	1.24 × 10^15^	-	22.0	-
-	-	-	-	1.23 × 10^15^	-	15.0	-
Moosrivier	16 January 2003	Unformulated	-	1.60 × 10^14^	0.5	35.0	Handguns
-	-	-	-	8.00 × 10^14^	-	-	-
Carden	3 December 2003	Cryptogran	10	6.59 × 10^13^	0.5	20.1 (STRB)^2^	Handguns
-	-	-	10	5.20 × 10^13^	0.25	15.3 (SCB)^3^	Oscillating tower
Bernol	1 December 2004	Cryptogran	10	4.49 × 10^13^	0.5	15.1	Oscillating tower
-	-	-	8	3.59 × 10^13^	-	-	-
-	-	-	6	2.69 × 10^13^	-	-	-
-	-	-	4	1.80 × 10^13^	-	-	-
-	-	-	2	8.98 × 10^12^	-	-	-
-	-	Cryptex	2.25	4.04 × 10^12^	0.5	-	-
Bernol	22 March 2005	Cryptogran	10	5.41 × 10^13^	-	19.5	Handguns
-	-	-	10	5.41 × 10^13^	0.5	-	-
-	-	-	10	5.41 × 10^13^	0.25	-	-
Dunbrody	26 October + 5 December 2006 + 5 February 2007	Cryptogran	10	6.37 × 10^13^	0.25	15.3	Oscillating tower
-	5 December 2006	-	10	6.37 × 10^13^	0.25	-	-
-	5 December 2006 + 5 February 2007	-	10	6.37 × 10^13^	0.25	-	-
Lone Tree	10 December 2007	Cryptogran	10	6.08 × 10^13^	-	21.9	Handguns
-	-	-	10	6.08 × 10^13^	0.25	-	-
Lone Tree	10 December 2008	Cryptogran	10	4.25 × 10^13^	-	21.9	Handguns
-	-	Cryptogran	10	4.25 × 10^13^	0.25	-	-
-	-	Cryptex	3.3	8.02 × 10^12^	0.25	-	-
Welgelegen	8 December 2009 + 15 March 2010	Cryptogran	10	2.80 × 10^13^	0.25	10.1	Oscillating tower
-	16 March 2010	Cryptogran	10	2.80 × 10^13^	0.25	-	-
Far Away	7 December 2010	Cryptogran	10	2.97 × 10^13^	-	10.7	Handguns
-	-	Cryptogran	10	-	0.25	-	-
-	-	Cryptex	3.3	3.91 × 10^12^	-	-	-
-	-	Cryptex	3.3	-	0.5	-	-
Bernol	19 December 2011	Cryptogran	10	6.66 × 10^13^	-	24.0	Handguns
-	-	Cryptogran	10		0.25	-	-
-	-	Cryptex	3.3	8.79 × 10^12^	-	-	-
-	-	Cryptex	3.3	-	0.5	-	-
Far Away	24 April 2013	Cryptogran	10	3.25 × 10^13^	0.25	11.7	Handguns
-	-	Cryptex	3.3	4.29 × 10^12^	-	-	-

^1^ Where molasses was added at 0.5%, no surfactant was added; where molasses was added at 0.25%, a surfactant was added; ^2^ STRB = single-tree randomised block design; ^3^ SCB = semi-commercial block.

### 4.6. Trial Evaluation

After application, all trials were evaluated in a similar manner. Fruit drop (from data trees) was evaluated from three weeks after application (unless stated otherwise), usually until harvest or until there was a substantial decline in efficacy. Evaluations were not initiated earlier than this, as experience has shown that infested fruit take a minimum of three weeks to drop off the tree [6]. Hence, evaluations conducted at three weeks after treatment were actually an indication of efficacy during the first week after spraying. In single-tree randomized block trials, each of the 10 to 12 trees per treatment was used as a data tree. For the semi-commercial block trials, five to 10 data trees were selected in the middle of each of the two replicate blocks (*i.e.*, a total of 10 to 20 data trees per treatment). Dropped fruit from each data tree were collected and analyzed separately. However, each data tree within each block could be considered as a pseudo-replicate. Fruit were analyzed by dissecting them carefully with a sharp knife and searching for any signs of larval infestation. Infested fruit were identified either by the presence of a *T. leucotreta* larva or its tunneling and frass [6], which are very characteristic of *T. leucotreta* infestation.

This fruit drop analysis protocol was considered to be the most accurate method for evaluating these trials, as all infested fruit would drop off the tree and it was therefore considered practically impossible to miss anything as a result of any sampling error or bias.

### 4.7. Data Analysis

Mean numbers of *T. leucotreta*-infested fruit per tree per week for each treatment (independently for each site) were compared over the full evaluation period in each trial, using a Generalized Linear Model ANOVA and the Fisher LSD multiple range test (or in one case the Students’ *t*-test), using Statistica 12.0 (Statsoft Inc., Tulsa, OK, USA, 2013). Weekly comparisons of means were not conducted, as this was considered superfluous compared to the comparison of means over the full period of evaluation.

## 5. Conclusions

In 13 field trials conducted between 2001 and 2013, CrleGV succeeded in reducing *T. leucotreta* damage by between 30% and 92%. These results were comparable with and sometimes better than those achieved with the chemical alternatives. The addition of molasses to sprays substantially and sometimes significantly improved efficacy. It is concluded that due to the efficacy of CrleGV, its almost unparalleled field persistence and its favorable non-target profile, it should form an integral part of a program for the control of *T. leucotreta* on citrus and other susceptible crops.
